# Carbon Nanotube and Its Derived Nanomaterials Based High Performance Biosensing Platform

**DOI:** 10.3390/bios12090731

**Published:** 2022-09-06

**Authors:** Jagannath Mondal, Jeong Man An, Sachin S. Surwase, Kushal Chakraborty, Sabuj Chandra Sutradhar, Joon Hwang, Jaewook Lee, Yong-Kyu Lee

**Affiliations:** 1 Department of Green Bio Engineering, Korea National University of Transportation, Chungju 27469, Korea; 2 Department of Bioengineering, College of Engineering, Hanyang University, Seoul 04763, Korea; 3 4D Convergence Technology Institute, Korea National University of Transportation, Jungpyeong 27909, Korea; 4 Department of IT and Energy Convergence (BK21 FOUR), Korea National University of Transportation, Chungju 27469, Korea; 5 Department of Aeronautical & Mechanical Design Engineering, Korea National University of Transportation, Chungju 27469, Korea; 6 Department of Chemical and Biological Engineering, Korea National University of Transportation, Chungju 27469, Korea

**Keywords:** carbon nanotubes, high-performance biosensors, nanomaterials-based biosensors

## Abstract

After the COVID-19 pandemic, the development of an accurate diagnosis and monitoring of diseases became a more important issue. In order to fabricate high-performance and sensitive biosensors, many researchers and scientists have used many kinds of nanomaterials such as metal nanoparticles (NPs), metal oxide NPs, quantum dots (QDs), and carbon nanomaterials including graphene and carbon nanotubes (CNTs). Among them, CNTs have been considered important biosensing channel candidates due to their excellent physical properties such as high electrical conductivity, strong mechanical properties, plasmonic properties, and so on. Thus, in this review, CNT-based biosensing systems are introduced and various sensing approaches such as electrochemical, optical, and electrical methods are reported. Moreover, such biosensing platforms showed excellent sensitivity and high selectivity against not only viruses but also virus DNA structures. So, based on the amazing potential of CNTs-based biosensing systems, healthcare and public health can be significantly improved.

## 1. Introduction

The detection of biological components is important in several areas ranging from healthcare, clinical medicine, environmental control, and food processing to homeland security [1,2]. Therefore, the development of reliable and cost-effective devices is highly demandable for our healthy lifestyles [3]. Sensor a class of devices that has been explored to detect a range of gas molecules to biomolecules. Biosensors are analytical devices that can combine biomolecules recognition via chemical or physical transduction [4]. Biosensor development is being driven increasingly by nanotechnology. Signal transduction is the basis for the operation of biosensors [5]. There are three elements in this system: a bio-recognition element, a bio-transducer, and an electronic system consisting of a display, a processor, and an amplifier. It interacts with a specific analyte through its bio-recognition element [6]. A wide range of samples can be tested with biosensors, including body fluids, food samples, and cell cultures. The main features of biosensors include: (a) being highly specific for the analyte, (b) reaction must be unaffected by factors like pH, temperature, or stirring, and (c) the linearity of the response will be maintained over a certain range of analyte concentrations [7,8,9].

One of the most widely used nanomaterials over the last two decades is carbon nanotubes (CNTs). This highly active field of research has a wide variety of CNT forms, and new forms are being designed and fabricated on a continuous basis. Hence, CNTs are receiving considerable attention in many fields of application from medicine [10], agriculture [11], and food safety [12] to bioprocessing [13], environmental [14], and industrial monitoring [15]. In recent years, CNTs have drawn interest in biosensor devices due to their several unique properties [16,17,18,19]. Specifically, CNTs possess a wide surface area and extensive free surface energy, and can easily stabilize the biomolecules at the biosensor surface through strong adsorb capability [20,21,22]. The strong carbon–carbon bonds and nanostructure of chemically modified carbon nanotubes (CNTs) are attributed to their outstanding electric conductivity, exceptional tensile strength, thermal conductivity, and optical properties allowing them to efficiently transmit signals associated with detecting analytes, metabolites, or disease biomarkers [23,24]. The use of CNTs for biomedical applications has therefore attracted considerable attention. Owing to their high surface-to-volume ratio, CNTs are capable of detecting biological components at ultra-fast speed with minimal concentrations. With the great advantages of CNTs-based biosensors such as high sensitivity, fast response time, lower potential for redox reactions, and longer lifetime with stability compared to other sensors based on metal oxides, or silicon-based materials [25,26]. These potential characteristics of CNTs have shed to elevate the research interests towards the development of biosensors. Electrochemical sensors and optical sensors made from CNTs have been developed for several applications, including the detection of heavy metals [27], in addition to field-effect devices for detecting virus infection [28], bacteria [29], cancer [30,31], diabetes [32], and biological components detection [33].

**Figure 1:**
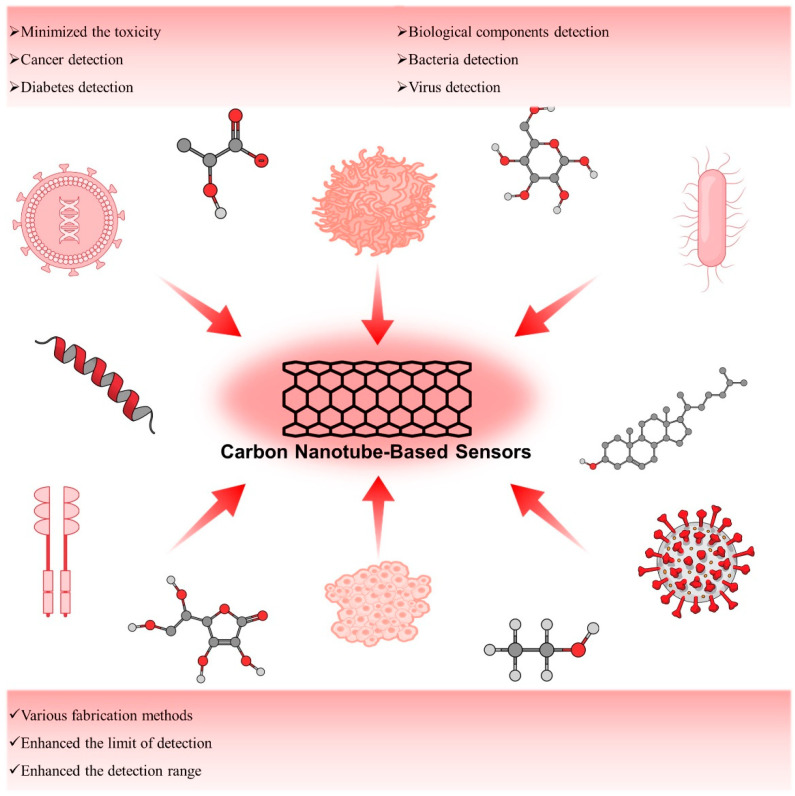
Schematic presentation of CNT-based biosensors.

The systematic review is an overview of carbon nanotubes (CNTs) and their derivatives as high-performance biosensors. The preparative methods such as electric-arc discharge, laser ablation, and chemical vapor deposition (CVD) of the CNTs have been described briefly here. The efforts made on the toxicology profile and mechanism of sensing of CNTs in this study. Later, we have illustrated insightfully the applications of CNTs as a biosensor for the detection of cancer and diabetes, biological components such as carbohydrates, proteins, essential elements, some bacteria, and viruses (Figure 1). Additionally, the recent development towards the commercialization of CNTs and their derivatives sensors has been discussed.

## 2. Preparation of Carbon Nanotubes (CNTs)

After the discovery of synthetic pathways of C_60_ and other types of fullerenes [34], it ignited the interest among synthetic chemists for other carbon-based materials with different structural possibilities. The first multi-walled CNT (MWCNT) synthesis in the laboratory was reported by Sumio Iijima in 1991 by using carbon black and graphite as precursor materials in a regulated environment. He named it “Helical microtubules” [35]. Iijima used the arc-discharge evaporation method to produce needle-like structures (ranging from 4–30 nm in length and up to 1 µm in diameter) comprising coaxial tubes of graphite sheets, but his unquenchable reaction setting was also associated with some major drawbacks including uneven shape, size, and mechanical strength as well as purity, which are the most important parameters associated with their applicability. In the case of single-walled CNT (SWCNT), it was jointly discovered by Iijima and Ichihashi [36] and Bethune and colleagues [37] in 1993. They used arc discharge methods to produce CNTs whereas the former group used an iron catalyst and the latter one used a cobalt catalyst. In both cases, uneven size was the biggest issue as Iijima and Ichihashi reported a diameter between 0.75 and 13 nm whereas Bethune and colleagues reported a diameter between 1.2 and 20 nm.

Thirty years after the first encounter, there is revolutionary progress in the field of CNTs. Currently, a variety of synthetic techniques are being employed with modified approaches and tweaks to produce CNTs with some exceptional features due to the recent revelation of CNT application in the pharmaceutical division. 

Electric-arc discharge, laser ablation, and chemical vapor deposition (CVD) are commonly used to produce several types of CNTs.

### 2.1. Electric-Arc Discharge

The oldest method of synthesis of CNTs is the arc discharge method. It utilizes the principle of breaking down the gas to generate plasma. The main component of this experimental setup is two parallelly attached electrodes (either horizontally or vertically) where the anode is crammed up with carbon precursors along with catalysts whereas the cathode is a pure graphite rod. The chamber is filled with an inert gas or engulfed inside a liquid atmosphere. Both AC and DC power supplies can be used in this system whilst the electrodes are kept in close contact (1–2 mm) to generate an arc and attain a steady discharge. Plasma is generated by arc current at an extremely elevated temperature (4000–6000 K) which sublimes the carbon precursor in the anode. The carbon vapors accumulate in the gaseous phase and get deposited at the cathode due to the temperature gradient. After cooling down it is taken out and purified for further investigation and examined under an electron microscope for further assessment of the morphology [38].

CNTs grow in different phases in this method as in the vapor phase, liquid phase, solid phase, and crystal phase, respectively [39,40,41,42]. 

In the arc discharge method, CNTs get synthesized by sublimation of a carbon precursor. Carbon black [43,44,45,46] and graphite [47,48,49,50,51,52,53,54,55,56] are commonly used in this scenario although some other carbon precursors namely fullerene waste soot [57], polyvinylalcohol [58], and other hydrocarbons including toluene, xylene, cyclohexane, cyclohexanone, n-hexane, n-heptane, n-octane, and n-pentane [59] are also reported. Some important parameters namely, the pressure of the inert gas, optimal voltage as well as the choice of the catalysts are critical for synthesizing highly pure CNTs via this method. For example, one of the recent findings suggests that the Co-MCM-41 catalyst helps to produce CNTs with large diameter distribution along with bulk production [60].

### 2.2. Laser Ablation

Laser vaporization or the laser ablation method is one of the most efficient methods to synthesize CNTs. It uses the same principle of arc discharge method as vaporization of carbon precursors in a laser-assisted pathway followed by depositing on the substrate. In 1995, R.E. Smalley and colleagues reported the first SWCNT synthesis by directly vaporizing transition metal/graphite composite rods in a laser-aided pathway. The experimental setup consists of a furnace, a quartz tube with a window, a target carbon composite doped with catalytic metals, a water-cooled trap, and flow systems for the buffer gas to maintain constant pressures and flow rates along with a pressure gas flow controller. Typically, an Nd:YAG (neodymium-yttrium-aluminum-garnet) laser or a CO_2_ laser is introduced through the window and focused onto the target. The target gets vaporized at a controlled pressure. The buffer gas transports the manufactured SWNTs towards the water-cooled trap, where they are collected [61]. In this method, it is possible to get a yield up to 90% but the high cost makes it pretty tough to implement in large-scale production. 

Arc discharge and laser ablation are both energy-intensive processes, therefore comparisons between them revealed some noteworthy commonalities. Such a situation is exceedingly uneconomical for performance at an industrial level. Both techniques have extremely rigorous purification protocols and huge graphite requirements as a target material, which restricts their use in large-scale industrial manufacturing.

### 2.3. Chemical Vapor Deposition (CVD)

The thermal CVD method is one of the simplest and most cost-efficient methods in the field of CNT synthesis which can produce a high amount of yield. CVD synthesized CNT was reported as defective at the beginning, but since 1998, after recognizing its potential, a significant number of changes have been incorporated and nowadays it is one of the most widely used methods for synthesizing CNTs. Currently, it is possible to engineer high-quality SWCNTs and MWCNTs via this method. One of the most important advantages it has over arc discharge and laser ablation methods is the temperature region as it can be operated in lower regions such as 550–1000 °C. First, the carbon source gas and the carrier gas are introduced into the reaction chamber while the temperature is between 550 and 1000 °C. Next, the gas is decomposed to produce carbon atoms on a coated catalyst substrate while the temperature is kept at a high temperature and finally carbon nanotubes are produced. The most common transition metal catalysts are cobalt (Co) [62], iron (Fe), nickel (Ni), copper (Cu), chromium (Mo), and their alloys [63]. In most cases, carbon gases such as methane, ethanol, ethylene, acetylene, and benzene are used as the source of carbon [64,65,66].

### 2.4. Others

Apart from conventional methods, there are also some other methods emerging for CNT synthesis, namely solvothermal [67], low-temperature plasma reduction [67], sol-gel [68], and flame [69] but low yield and difficult parameter control make it very hard for them to get used in frontline synthesis. 

## 3. Mechanism of Sensing

Due to excellent mechanical, electrical, and electrochemical properties, CNTs are widely used in the field of biosensing. In 1962, Leland C. Clark, Jr, and Champ Lyons published a report regarding the electrode system for cardiovascular surgery [70]; this is the central principle on which CNT-based biosensors rely. It has been found that SWCNTs have a broad array of electrical conductivity properties coupled with high chemical stability and extraordinarily hefty length to diameter ratios, which may be as high as 132,000,000:1. While metallic SWCNTs are also well suited for electrochemical implements, MWCNTs have some advantages due to their superior metallic electronic properties [71]. CNT-based biosensors have two main components: a biological sensitive element and a transducer. CNTs are subjected to chemical alteration techniques to make them biologically responsive, and the transducer then converts the concentration of the analyte to other detectable physical variables such as current, absorbance, etc., which further can be analyzed and assessed [72]. The sensing principle can be divided into two categories, physical and chemical. The operating principle of physical sensing mimics the human response towards external stimuli, it works by analyzing the response of the device that bids reaction to the physical possession of the medium, and thus shows the “physical biosensor” term was coined whereas the principle of chemical sensing relies on the measurability of analyte concentration.

### 3.1. Physical Sensing

Physical biosensors can be classified into three categories: optical, piezoelectric, and calorimetric. For optical sensing, SWCNTs are specifically utilized as different chiralities have shown different electronic properties, exploiting those traits metallic, semi-metallic and semiconducting CNT-based biosensors are produced. Already there is a strong relationship being drawn between electronic structure and its photophysical behavior, which is crucial for advancement in this section. Michael J. O’Connell and colleagues have reported the fluorescent property of semiconducting SWCNTs in the near-infrared (NIR, 900–1600 nm) spectrum due to their electronic band gap between valence and conduction band, as well as a drastic reduction in photoluminescence intensity due to the aggregation phenomenon of isolated nanotubes [73]. The course of mechanisms of fluorescence modulation in CNTs can be depicted using several different shades namely Solvatochromism, charge transfer, and doping and redox reactions. Creating a microenvironment of nonpolar solvent around SWCNTs can significantly alter the photophysical properties resulting in a solvatochromic shift ranging from 25 to 100 meV [74]. In the case of photo-induced charge transfer, the plausible mechanistic pathway follows the ground-state thermal charge transfer from the nanotube valence band and photo-induced excited-state charge transfer from the nanotube conduction band resulting in quenching in fluorescence spectra [75]. In the case of doping, the analyte adjusts exciton decay paths by altering the number of carbon lattice defects whilst the redox reactions are responsible for brightening the fluorescence [76] as well as reversible quenching [77,78]. Another important feature of SWCNT is the resonance-enhanced Raman signature, which is also used for advanced biosensing devices [79]. As for piezorestivity, that is due to CNT’s high strength, toughness, and exceedingly high Young’s modulus which is more than 1 TPa [80]. For detection, a high signal-to-noise ratio is essential for field-effect transistors. From Thomas Helbling and colleagues’ report, where they investigated the signal-to-noise ratio in carbon nanotube piezoresistive transducer elements and drew the correlation between the signal-to-noise ratio of SWCNT and gate bias voltage and concluded that the best operating strategy of SWCNT-based biosensors will be at device off-state [81]. For the calorimetric CNT-biosensor, it operates by monitoring the temperature change and heat effect after the reaction of immobilized biological materials with corresponding objects which could be detected by the transducer [23].

### 3.2. Chemical Sensing

According to the IUPAC definition, a self-contained integrated analytical device can be called an electrochemical biosensor when it uses a biological recognition element (biochemical receptor) that is kept in close proximity to an electrochemical transduction element to provide exact quantitative or semi-quantitative analytical information. They are clearly distinguishable from a bioanalytical system which necessitates additional processing steps, such as reagent addition [82]. Electrochemical biosensors based on CNTs can be classified into three categories namely, amperometric, potentiometric, and impedimetric biosensors, and among them, the amperometric method is most extensively used. The main principle of this type of sensing is to change the chemical signal to electrical signals, once the enzyme electrode is submerged in the test solution, the analytes will diffuse into the enzyme layer and quickly undergo an enzymatic reaction. Amperometric CNT-based biosensors work by using an enzyme-fixed CNT electrode to catalyze oxidation or reduction as the sensing mechanism. Following this principle, different types of different enzyme-based electrodes are reported to date including nicotinamide adenine dinucleotide (NADH) [83], glucose oxidase (GOD) [84], lactic acid oxidase [85], cholesterol oxidase [86], etc. In the case of potentiometric sensors, transducers measure potential based on the intensification of charges on the working electrode, where biochemical receptors converse with analytes. When negligible current flows between the reference and working electrodes, the potential is recorded between them. Potentiometric devices typically translate the ion activity signal during electrochemical feedback [87]. The impedimetric sensing pathway is further classified into two, faradaic and non-faradaic. In the faradaic pathway, the electrochemical transducer, over a wide range of applied alternating current frequencies, measures the resistance and reactance produced by the charge transfer aptitude of an analyte between an electrode and a redox electrolyte solution or redox medium between electrodes or a reference node whilst for the non-faradaic pathway, the change in dielectric parameters namely capacitance, impedance, permittivity, and current in between the electrode and the medium (electrolyte) as well as at the sensor electrode interface are directly measured [88].

## 4. Toxicology Profile of Carbon Nanotubes

Nanomaterials for therapeutic and sensor applications have non-negligible toxicity. It is caused by a very large surface area, the inherent toxicity of the material itself, and its very small size [89]. In addition, it is very important to determine the toxicity of carbon nanotubes for clinical use because there is a possibility of accumulation in organs [90]. Like many studies conducted for clinical application, CNTs also need to be carefully evaluated and verified for toxicity in vitro and in vivo [91,92]. Here, we provide the toxicity of carbon nanotubes and present methodological explanations to reduce toxicity. Epithelial cell proliferation is a general phenomenon that occurs in recovery after tissue damage caused by foreign substances. This is particularly noticeable in CNT-treated lungs [93]. CNTs at a concentration of 6 mg/m^3^ were inhaled by rats for 13 weeks. After 39 weeks, bronchiolar and alveolar hyperplasia were confirmed [94]. However, no cell hyperproliferation was found in mice that inhaled CNTs at low concentrations (1.5 mg/m^3^). Therefore, exposure to CNTs can induce fibrosis by irritating the respiratory system, such as bronchioles and alveolar ducts, depending on their concentration. Repetitive stimulation of cells results in genetic damage, which causes a very fatal problem in cell proliferation and division [95]. Recent studies have shown that multi-walled carbon nanotubes (MWCNTs) induced DNA breaks and mutations in cells of the respiratory system as well as being acute genotoxic [96]. MWCNT showed DNA strand damage in vitro in human bronchial epithelial BEAS-2B cells. MWCNTs also showed an in vivo dose-dependent increase in damage of DNA strands with single pharyngeal aspiration in the lung cells, bronchoalveolar lavage cells, and micronucleated alveolar type II cells. MWCNTs caused DNA damage after inhalation but not after pharyngeal aspiration in bronchoalveolar lavage cells. This phenomenon possibly reflects changes in the BAL cell population following the bolus dose. Their findings indicate that straight MWCNTs induce not only DNA damage in vitro but also produce both DNA damage and micronuclei in mouse lungs. The toxicology results using rats (Sprague–Dawley rats) are similar to the mice used in research [97]. MWCNTs were administered intratracheally (0.5, 2, or 5 mg) to Sprague–Dawley rats, and inflammation and fibrosis level were evaluated. MWCNTs were still not only present in the lung after 60 days but also induced inflammation and fibrosis. MWCNTs lead to a significantly higher level of TNF-a and the formation of collagen in the lung. Therefore, due to the accumulation of CNTs in the tissue, the tissue gets damaged and stimulation lasts for a long time, which not only increases the secretion of inflammatory cytokines but also causes genetic damage. These continuously occurring actions eventually lead to fatal function loss of organs and act as a major hurdle for the clinical application of CNTs.

The liver is one of the organs most closely related to metabolism [98,99]. Metabolism and absorption of nutrients, drugs, and other foreign substances occur mainly in the liver [100]. In particular, since it plays an important role in the metabolism and detoxification of drugs, it is essential to check whether toxicity occurs in the liver for the clinical use of CNT [101]. CNT will accumulate in the liver and should be closely monitored to see if this causes toxicity problems [102]. Zongfei Ji et al. reported severe hepatotoxicity of MWCNT [103]. The two types of MWCNT (acid-oxidized MWCNT and Tween-80-dispersed MWCNT) were intravenously injected to investigate hepatotoxicity. The body weight of the mice injected with MWCNT was reduced and the color of the liver changed to dark red. In addition, the level of total bilirubin was increased. Bilirubin is one of the components of bile and is produced from hemoglobin [104]. Red blood cells composed of hemoglobin circulate throughout the body for about 3 weeks and undergo gas exchange in the tissues, and then are destroyed. At this time, red blood cells are metabolized to form bilirubin. The produced bilirubin is secreted together with bile from the liver, decomposed in the small intestine, and finally excreted from the body. However, the concentration of bilirubin in the blood increases when the metabolic process does not proceed smoothly due to liver damage or decreased function [105]. With the increased concentration of bilirubin, liver function and damage can be determined. Zongfei Ji et al. investigated Kunming mice and reported a significantly increased (2-fold increased, 0.5 mmol L^−1^ to 1.0 mmol L^−1^) bilirubin level in the MWCNT-treated group which was exposed to 10 and 60 mg/kg by intravenous injection for 15 and 60 days. The result also reported a significantly increased (27% increased, 110 U L^−1^ to 140 U L^−1^) aspartate aminotransferase level. Aspartate aminotransferase is an enzyme that can detect liver disease [106]. Aspartate aminotransferase exists in hepatocytes and is basically detected in the blood. However, when abnormal liver function, degeneration and destruction of liver tissue, and various liver diseases occur, aspartate aminotransferase is released from the liver into the blood, leading to changes in concentration in the blood [107]. Through this change in blood concentration, liver disease and liver damage can be checked. As well as the problems of long-term accumulation in the lungs mentioned earlier, they reported the accumulation of MWCNTs in the liver up to 60 days after administration and histological morphology changes in livers and inflammatory infiltration. Moreover, when the liver tissue was observed with a transmission electron microscope (TEM), it was confirmed that the mitochondria were physically destroyed due to the penetration of MWCNTs. These results suggest the possibility that MWCNTs physically destroy mitochondria, creating additional problems beyond the toxicity discussed so far.

Through the process of decomposing and excreting most foreign substances including CNTs, the body protects itself and maintains homeostasis [108,109,110]. We also need to look closely at the toxicity of CNTs to the excretory organs that are responsible for the clearance of drugs and foreign substances. The kidney is a very important organ for the excretion of toxins, and it is necessary to accurately study whether CNTs accumulate in the kidney during excretion and whether toxicity is caused by CNT. Zamani F et al. used MWCNTs for the construction of an MWCNT-induced kidney injury model [111]. They reported the assessment of apigenin against an MWCNT-induced kidney-injured rat model. As a flavonoid, apigenin is contained in a large amount in various fruits and vegetables [112], and has the effect of suppressing inflammation [113], oxidative stress [114], and ameliorates carbohydrate metabolism [115]. They administered 10 mg kg^−1^ of apigenin to Wistar rats. After 2 weeks, the Wistar rats were exposed to MWCNTs for 5 h/day and 5 days/week. After exposure to MWCNTs, mitochondria were extracted from the kidneys of Wistar rats and mitochondrial toxicity parameters were analyzed to evaluate the renal toxicity of MWCNTs. Succinate dehydrogenase is an important parameter that can be used to evaluate kidney-derived mitochondrial toxicity. Succinate dehydrogenase is found in almost all cells. In animal cells, it is present in the mitochondria and is contained in the TCA cycle. It is an important enzyme that is particularly closely related to the respiratory chain [116]. The main role of this enzyme is to catalyze fumarate production by dehydrogenation of succinate [117]. Surprisingly, the activity of mitochondrial succinate dehydrogenase from kidneys that are harvested from MWCNT-exposed Wistar rats was significantly reduced to 25% compared to the control group. In addition, mitochondria in the kidneys of the MWCNT-treated group swelled by nearly 50%, and the number of reactive oxygen species was increased by 2.5 times. They also investigated the release of cytochrome c. Cytochrome is a hemeprotein that plays a role in the transport of electrons as an electron transporter [118]. Among the hemeproteins present in cells except for myoglobin, peroxidase, and catalase are cytochromes and those are classified into various types such as cytochromes a, b, and c. When apoptosis proceeds, cytochrome c inside the mitochondria is released [119]. For this reason, it is possible to determine whether apoptosis is progressing based on the released cytochrome c. Results showed that cytochrome c was released more than 2-fold as compared to the control group from rat kidney mitochondria after the inhalation exposure to MWCNTs. In conclusion, they reported that MWCNTs induce nephrotoxicity and apigenin exerted its protective effect through the reduction in ROS-mediated oxidative stress and mitochondrial damage.

The spleen is the largest secondary lymphoid organ in the human body and plays the most important role in eliciting and regulating the immune response for the removal of foreign substances [120,121]. The spleen, like the liver, lungs, and kidneys, is one of the organs most likely to be damaged due to its high sensitivity to foreign substances. In addition, the spleen is more burdened by the removal of foreign substances through the immune response. When a toxic substance enters the body, an inflammatory response is triggered, and lymphatic organs including the spleen and immune cells are activated to respond to the inflammatory response [120,122]. Abnormalities of the immune system affect the whole body as well as the lymphatic organ. Therefore, immune system toxicity of CNT is important for further application. Lee S et al. reported that less-dispersed single-walled carbon nanotubes (SWCNTs) caused cytotoxicity in macrophages and abnormalities in immune organs such as the spleen [123]. As a result, treatment with 10 µg mL^−1^ of SWCNTs in J774A.1, a rodent macrophage cell line, significantly decreased the cell viability to 30% after 24 h. In addition, the concentration of reactive oxygen species in the cells increased more than 4 times, and the concentration of superoxide dismutase was increased by 2.4 times. In the cells treated with SWCNTs, the concentrations of inflammatory cytokines, IL-1β and TNF-α, also showed a 2-fold increase. To investigate the effect of SWCNTs in vivo, BALB/c mice were intravenously injected with SWCNTs (1 mg kg^−1^ every day) for 2 weeks. The spleen was stimulated by SWCNTs, and splenocyte proliferation was significantly increased by more than 2-fold. Concentrations of inflammatory cytokines in the BALB/c were examined, and IL-2, IL-1β, and TNF-α also showed significant increases. Clichici S et al. reported the short-term impact of MWCNTs on the spleen [124]. They produced DNA functionalized MWCNT to improve the dispersity of MWCNT in the body. MWCNT were intraperitoneally administrated to Wistar rats (single dose; 270 mg L^−1^ of MWCNT). MWCNT were translocated into the spleen, showing maximum concentration at 48 h after administration. Through histological analysis, a large number of macrophages were identified in the spleen of the MWCNT-administered group, and it was confirmed that apoptosis also occurred actively. Oxidative stress was further increased as the concentration of nitric oxide (3-fold) and inducible NO synthase (2.5-fold) in the spleen increased. The concentration of interleukin-1b (one of the pro-inflammatory cytokines which is an important biomarker for assessing the level of the inflammatory response [125]) and caspase-3, proliferating cell nuclear antigen (PCNA)-expressing cells. The concentration of interleukin-1b increased more than 4 times, and the number of cells expressing caspase-3 increased by 2.3 times. In addition, the number of PCNA-expressing cells increased by 1.6 times. Eventually, the spleen swelled, and the weight increased from 0.68 ± 0.26 g to 0.87 ± 0.29. Since this study is a short-term study that evaluates toxicity with a single dose, additional long-term toxicity studies are required.

For further applications, developments, and clinical use, the safety of CNTs would be established carefully. Various studies have been conducted to eliminate the toxicity of CNTs. A method to reduce toxicity through modification of CNT with PEG has been proposed [126]. PEG is a biocompatible polymer, and PEG is already widely used in other drugs, diseases, and various fields [127]. Researchers modified PEG on CNT (pegylated CNTs) and looked at their toxicity in the liver [126]. Single-walled CNTs (SWCNTs) and PEG-SWCNTs were examined and both CNT were retained in the liver for more than 4 months. However, there was no statistically significant change in body weight and blood pressure between the control and CNT-treated groups. The serological tests and biochemical inflammatory factors levels in the CNT-treated group support that PEG-SWCNT are not toxic material for the body. Many studies have been conducted to reduce the toxicity in vivo application of CNTs through many approaches such as not only pegylation but also hydroxylation [128], carboxylation [129], amination [130], surface coating [131], and antibody binding [132]. Liu Z et al. compared the cytotoxicity of hydroxylated multi-walled carbon nanotubes (MWCNTs–OH) on L02 cells for 24, 48, and 72 h with pristine multi-walled carbon nanotubes [128]. They also measured the mitochondrial membrane potential in L02 cells. Furthermore, the effects of MWCNTs-OH on the activation of caspase-3 and caspase-9 (which have distinct roles during intrinsic apoptosis [133]) in L02 cells for detailed examination of the apoptosis process were also assessed. They discovered that MWCNTs–OH triggered a significantly milder cytotoxic response than that of pristine multi-walled carbon nanotubes. The results further showed that such an attenuated response could be attributed to a reduced disruption of the mitochondrial membrane potential. MWCNTs–OH also leads to the attenuation of both cytochrome c release from mitochondria to cytoplasm and activation of caspases-3 and caspases-9. These results show that CNTs whose surface has been modified with hydroxyl groups have statistically significantly lower toxicity to cells than pristine multi-walled carbon nanotubes. This means that, as the previous results showed, CNTs whose surface was modified with hydroxyl groups are less likely to disrupt the mitochondrial surface and cause less apoptosis. Sweeney S et al. investigated the physicochemical characteristics and toxicity of two MWCNT materials: acid purified ‘Purified-MWCNT’ and concentrated acid functionalized ‘COOH-MWCNT’ [129]. Purified-MWCNT were significantly more toxic as measured by reduced alveolar macrophage viability and increased inflammatory mediator releases such as IL-1β and IL-8. Those cytokines were released 3.5- and 2.4-fold more respectively by alveolar macrophages after 24 h of purified-MWCNT treatment. In contrast, those cytokines released by alveolar macrophages did not significantly change after 24 h of COOH-MWCNT treatment. Therefore, the secretion of inflammatory cytokines does not increase significantly because COOH-MWCNTs do not vigorously stimulate alveolar macrophages and are relatively less recognized as toxic substances by alveolar macrophages. This result means that COOH-MWCNTs are less toxic than purified-MWCNTs and that when applied to the human body, toxicity due to immune response is significantly lower. For the clinical use of CNTs, it is necessary to consider a method for modifying CNTs that is appropriate according to the target disease, type of drug, and route of administration.

## 5. Applications of Carbon Nanotubes as Biosensors

### 5.1. Carbon Nanotube-Based Sensors for Detection of Cancer

#### 5.1.1. CD44 Expressing Cancer Cell

**Figure 2:**
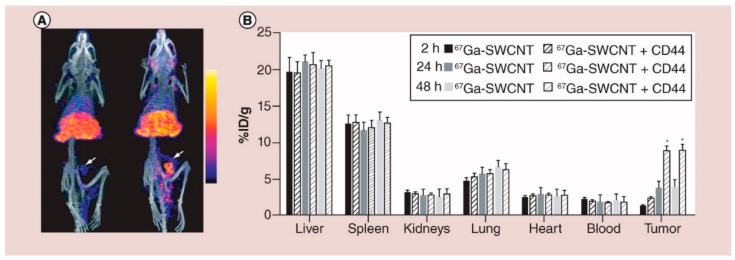
Biodistribution of Gallium-67-labeled single-walled carbon nanotubes in murine breast cancer model. (**A**) SPECT/CT images of tumor-bearing mice after 24 h of injection of either Ga-SWCNT or Ga-SWCNT + CD44 (right). (**B**) Quantitative measurement of radioactivity of Ga-SWCNT or Ga-SWCNT + CD44 at various organs and tumor after dosing at different time points: 2, 24, and 48 h represented as dose per gram of tissue. Image reproduced with permission from [134]. Copyright 2016 Future Medicine Ltd.

Recently, cancer stem cells (CSCs) were identified as rare tumor-initiating cell populations which show self-renewal, pluripotent, and highly tumorigenic which makes them more resistant to breast cancer treatment. These cells are mainly responsible for breast cancer recurrence since even though most of the cells were killed by therapy, still few CSCs can regenerate tumors. Notably, CSCs are isolated from various cancer types including breast, brain, lung, colon, and skin cancer or melanoma. In particular, the case of breast cancer CSCs is identified by the presence of characteristic biomarkers namely CD44 and CD24 as well as one of the enzyme activities (ALDH1). This literature suggests that it is very important to detect and target the CSCs and their daughter cells responsible for cancer regeneration to achieve double remission. Al Faraj et al. reported an efficient nanoprobe based on functionalized cancer nanotubes that can selectively detect breast cancer in the murine model as shown in Figure 2.

In this study, a novel and biocompatible nanoprobe was developed to selectively target the CSCs cells responsible for the regeneration of breast cancer while allowing to monitor the biodistribution of a probe with noninvasive imaging techniques after I.V administration of a carbon nanotube-based nanoprobe. To construct the nanoprobe, PEG-conjugated SWCNTs were functionalized with anti-CD44 antibody capable of CSCs detection at the breast cancer tumor site. This probe allowed to monitor biodistribution with various non-invasive modalities such as MRI/SPECT/NIR fluorescence imaging.

#### 5.1.2. EpCAM Expressing Cancer Cell

**Figure 3:**
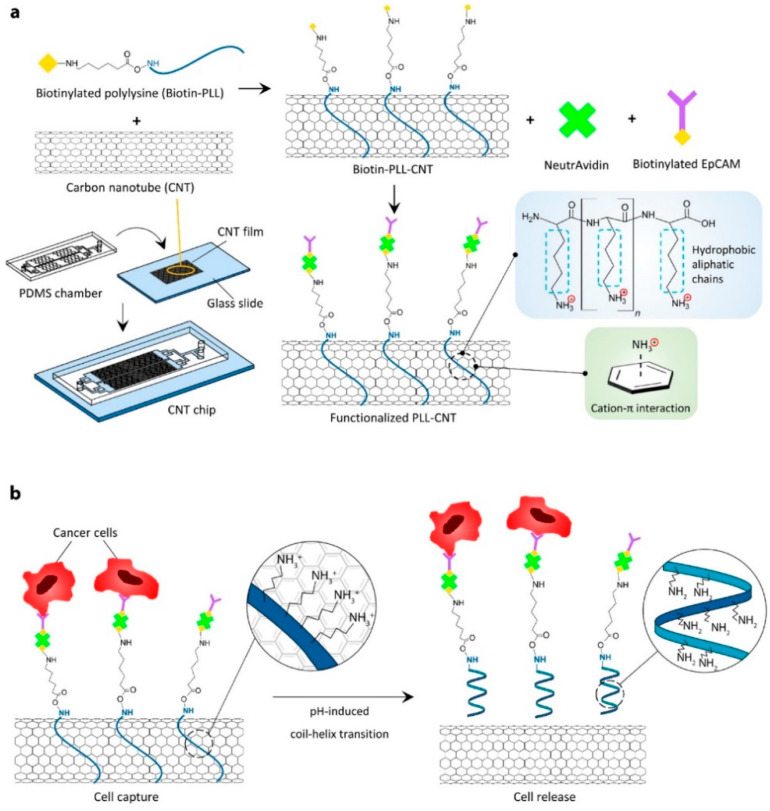
(**a**) Functionalization and fabrication of CNTs chip. In order to fabricate the chip CNT film was allowed to adsorb on the glass substrate followed by sealing with PDMS cover. Biotin-PLL was attached to the CNT film via Pi and hydrophobic interaction. (**b**) Schematic illustration showing the release mechanism of captured CTCs. There will be deprotonation of positively charged amino groups causing the conformational changes in the PLL structure of random coil to alfa-helix resulting in cancellation of interaction between PLL and CNTs releasing the captured CTCs. Image reproduced with permission from [135]. Copyright 2022 American Chemical Society.

Epithelial cell adhesion/activating molecule abbreviated as EpCAM is the first tumor-associated antigen and currently it is considered as the most intensely and frequently expressed tumor-associated antigen. It is found to be expressed in a great variety of cancer types and it can be utilized as a biosensor application. Circulating tumor cells (CTCs) can also express EpCAM antigen and a nanoprobe or sensor can be developed to study the presence of these biomarkers on cancer cells by using anti-EpCAM antibody. Neoh et al. developed a CNT chip containing promising microfluidic technology for the effective capture and release of the CTCs. This technique allowed to perform downstream analysis of CTCs such as molecular and functional analyses as shown in Figure 3. Researchers successfully developed a chip platform with the ability to not only capture the CTSs but also release them in a pH-responsive manner with higher sensitivity. This platform was tested for the clinical samples for the optimization of a device in order to maximize the cell capture and release efficiency, viability as well as application of this technology for single-cell molecular profiling and in vitro culture. Since EpCAM is a widely expressed antigen by various cancer cells, this platform could be generalized for different types of CTCs capture and detailed analysis.

#### 5.1.3. CA19-9 Expressing Cancer Cell

**Figure 4:**
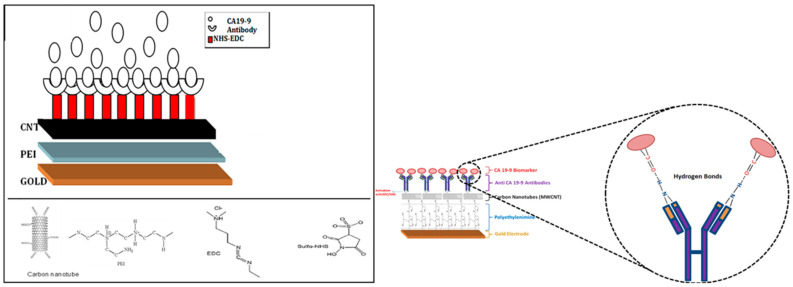
Using CNTs and PEI layer-by-layer (LbL) assembly was constructed on gold surface to fabricate the thin film. Carboxylic acid groups on the CNT surface were activated using EDC-NHS reagents to attach the anti-CA19-9 antibodies. Right side image shows the antibody–antigen reaction. Image reproduced with permission from [137]. Copyright 2017 American Chemical Society.

Carbohydrate antigen 19-9 (CA 19-9) is a cell protein glycoprotein also known as Sialyl Lewis-a produced by ductal cells in the pancreas, salivary gland, biliary system, and epithelial cells in the stomach and colon. It is the most used biomarker for the diagnosis and management of prognosticating pancreatic ductal adenocarcinoma (PDAC) [136]. Its widespread expression in several tumor cells makes it useful for the diagnosis of other tumor types apart from its historical use in the case of PDAC. Considering its diagnostic potential Thapa et al. developed a highly sensitive biosensor to detect the pancreatic cancer biomarker CA19-9. This developed biosensor based on nanomaterials promises cheaper, faster, and more efficient early diagnosis of pancreatic cancer as compared to traditional bulky devices. To fabricate the device, MWCNTs with functionalized anti-CA19-9 antibody were utilized. As shown in Figure 4, a concentric bundle of carbon nanotubes to construct the thin film was used to make the sensing part of the biosensor device. Functional carboxyl groups on the surface of functionalized CNTs act as antibody attachment sites. MWCNTs are particularly important for immunosensors since they can provide a large number of sites for antibody binding in the active layer of the sensor.

In this study, a sensitive biosensor based on impedance spectroscopy was designed and the selectivity of the biosensor towards the CA19-9 was confirmed by checking various other biological samples containing glucose, ascorbic acid, and antigen p53. To check the selectivity between samples with and without CA19-9 biomarkers, a multidimensional projection technique along with computer software was used. This biosensor showed promising results in terms of predicting the pancreatic cancer probability among various blood samples from patients.

#### 5.1.4. VEGF Expressing Cancer Cell

**Figure 5:**
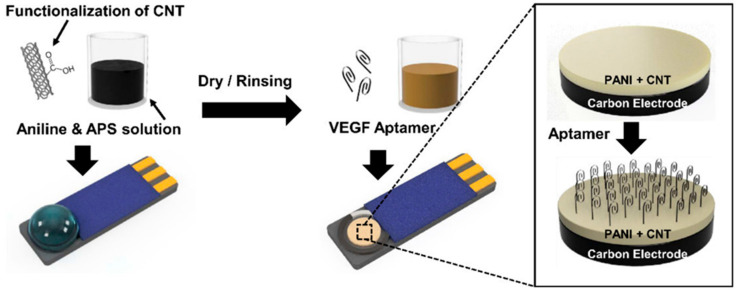
Schematic illustration showing the fabrication of nano-sensor device: preparation of nanocomposite of PANI/CNT and assembly of aptamer VEGF on the sensor surface. Image reproduced with permission from [139].

Vascular endothelial growth factor (VEGF) is considered a main angiogenic factor in the case of many malignant tumors. VEGF acts by specific effects by stimulating cell growth and migration as well as increasing vascular permeability. It is a promising biomarker, especially in the case of the prognosis of cancer cells [138]. Electrochemical aptasensors are promising agents due to their advantages of being cheaper and the possibility to have quantitative analysis. Park et al. [139] developed a stable and sensitive sensor based on polyaniline (PANI) and carbon nanotube (CNT) nanocomposites which uses anti-VEGF antibody to detect cancer cells as shown in Figure 5.

Recently, aptamers have emerged as sensor agents due to their higher binding ability and stability. Aptamers are either artificial oligo-nucleic acid or peptide molecules that act as single-stranded DNA (ssDNA) or RNA. Aptamers are considered “artificial antibodies” which can replace animal-derived antibodies and have the advantage of being easy to synthesize, low cost, and stable as compared to animal-derived antibodies. PANI is an attractive conducting polymer with great potential to use in sensor applications when combined with nanomaterials such as CNTs. Combining the PANI with CNTs helps to increase the surface-to-volume ratio. Additionally, this composite of PANI/CNT can provide higher sensitivity as well as minimizes the signal intensity by providing the direct path for transportation of charge. This composite has assured potential to be used in a flexible device due to the polymeric nature of the PANI.

#### 5.1.5. MUC−1 Expressing Cancer Cell

**Figure 6:**
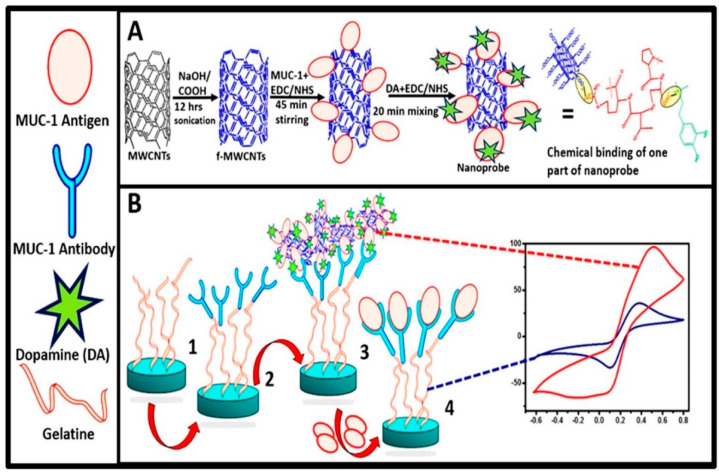
(**A**) Schematic illustration of the nanoprobe fabrication steps. (**B**) Working electrode modification along with immunosensor principle based on direct competitive electrochemical sensor for MUC−1 detection. Several steps are involved: (1) Electro oxidative grafting of gelatin on electrode; (2) MUC−1 antibody binding with EDC/NHS; (3) attaching nanoprobe with modified electrode; (4) decrease in signal after free MUC−1 replaced nanoprobe in a competitive assay. Image reproduced with permission from [140]. Copyright 2020 Elsevier.

A broad range of human epithelia express mucin 1 (MUC−1) including gastric, colorectal, and lung cells. Overexpressed MUC−1 is related to various cancers such as stomach, lung, and breast cancer. The exact detection of MUC−1 is very crucial for the early detection of cancer cells. Aptamers can be used to detect MUC−1 overexpression at greater sensitivity and stability while combined with carbon nanotubes. Rashid et al. fabricated a nanosensor for the detection of MUC−1 expression on cancer cells as shown in Figure 6. In this study, they tried to explore the advantages of aptamer combined with the CNTs to fabricate the nanoprobe. Additionally, dopamine was used to enhance the sensitivity of the nanoprobe because of its hydrophilic nature along with electron donors with variable redox properties. The inclusion of DA resulted in the generation of a signal amplification probe. As shown in Figure 5, in order to construct the nanoprobe, DA was linked to the MWCNTs using its carboxylic group, and MUC−1 was linked to MWCNTs with the help of an amine group. This probe was integrated with an electrode to form the competitive electrochemical immunosensor. In the case of ultrasensitive immunosensors, nanomaterials are incorporated to serve as an electro-active label or as a substrate to immobilize electro-active agents. MWCNTs are considered an important nanomaterial to increase the electron transfer rate. Additionally, due their intrinsic electrical and electrochemical properties, they are highly promising in sensing strategies.

Combining the Ag or DA along with fMWCNTs offered an advantage of higher sensitivity over the reported electro-active labels. Modification of carbon nanotubes with compatible polymers helped to reduce toxicity and biological damage. These modifications provided an ideal and conducive platform because of the amino-carboxyl surface chemistry of gelatin and fMWCNTs. This type of immonosensor can be easily adapted for the clinical diagnosis of various cancer types by changing the aptamer or conjugates antibody for the detection of biomarkers. Immunosensors based on nanomaterials could be useful for the early detection as well as monitoring of the disease progression. Table 1 shows a summary of CNTs and their derivatives as biosensors for cancer detection.

### 5.2. Carbon Nanotube-Based Sensors for Detection of Diabetes

Two primary approaches are used while incorporating nanotechnology for glucose sensing applications. In the first approach, sensors can be designed by using micro or macroscopic components while incorporating nanomaterials in the sensing device. These nanomaterials in the sensor design offer several advantages such as higher surface area and enhanced catalytic activity. In the case of the second approach, nanofabrication can generate nanoscale sensors for glucose sensing. These sensors have some advantages such as offering continuous monitoring and avoiding foreign body responses of the immune system resulting in a longer life as compared to traditional sensors. In the case of diabetes, CNTs incorporation is heavily investigated as enzymatic electrode detection of glucose due to the electron transfer ability of the CNT and their surface areas [146]. CNT-based electrochemical biosensors immensely helped with glucose sensing. Both single-walled CNTs as well as multi-walled CNTs have been explored as a nanomaterial for the detection of glucose. Functionalization of MWCNTs is less complex as compared to the SWCNTs since GOx could be directly adsorbed on the surface of MWCNTs as compared to the SWCNTs where a covalent linkage is required. It is possible to fabricate the best-performing glucose sensing devices when they are combined with the other nanomaterials [147].

Enzymatic sensors are based on the use of enzymes for the conversion of an electro-inactive substrate into an electro-active product such as the use of glucose oxidase enzyme on a platinum electrode. On the other hand, non-enzymatic glucose sensors are based on the direct electrochemical oxidation of glucose. Most of the researchers focused on the development of enzymatic electrochemical sensors using glucose oxidase, but recently, non-enzymatic sensors using direct electrochemistry of glucose on noble metals are coming forward as next-generation glucose sensing technology [148]. Here are examples of enzymatic and non-enzymatic sensors fabricated to enhance the ultrasensitive detection of glucose. Researchers tried to use carbon nanotubes to either modify the sensitivity of enzyme-based sensors that are prone to temperature-based degradation or used an alternative non-enzymatic sensing strategy by combining the carbon nanotubes in the device fabrications. Additionally, we have presented a tabular form of CNT-based enzymatic and non-enzymatic biosensors (Table 2).

#### 5.2.1. SWCNTs Based Nanosensors

**Figure 7:**
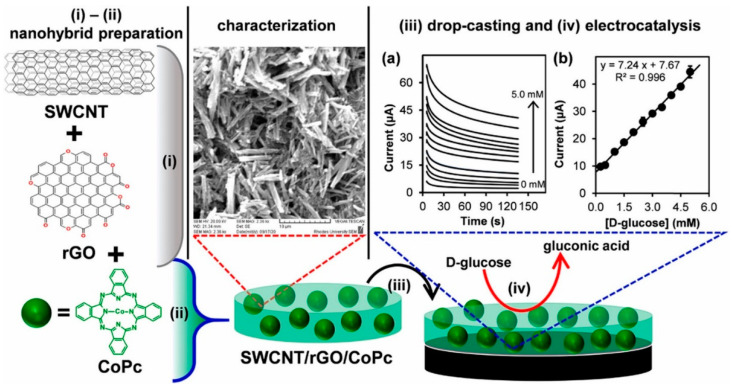
(i, ii) Nanohybrid preparation: SWCNT was modified with reduced graphene oxide (which was prepared by Improved Hummers Method) and cobalt phthalocyanine. The SEM image corresponds to SWCNT/rGO/CoPc nanohybrid. (iii) Fabrication of sensing electrodes (glassy carbon electrode or GCE) by drop-casting method. (iv) Electrochemical oxidation of D-glucose to gluconic acid. (a) Static chronoamperogram response of GCE-SWCNT/rGO/CoPc with increasing glucose concentrations from 0 mM to 5.0 mM in 0.10 M NaOH, and (b) the corresponding calibration plot of steady-state current against concentrations of glucose.. Image reproduced with permission from [182]. Copyright 2021 Elsevier.

The development of a non-invasive, cost-effective, and painless glucose monitoring system would be very helpful for diabetes patients. Detection of glucose in the saliva can be an ideal biological fluid providing non-invasive glucose monitoring as well as ease of sample collection. However, saliva glucose monitoring is considered challenging due to lower glucose concentration. Even though saliva is the most suitable non-invasive fluid and has a better correlation with blood glucose levels, it still needs to employ ultrasensitive glucose-sensing devices. Most of the currently reported glucose sensors based on GOx have limitations due to intrinsic limitations of enzyme GOx. An alternative can be the use of non-enzymatic glucose sensors, but they also have several drawbacks mainly associated with the cost of noble metals which makes these sensors very expensive. Recently, researchers reported that the use of carbon nanotubes (CNTs)/reduced graphene oxide (rGO) can greatly help for the synergistic improvement in electrochemical properties along with better analytical performance. Adeniyi et al. [182] prepared a nanohybrid electrocatalyst-based sensor for the ultrasensitive detection of glucose in saliva. To do so, they utilized SWCNTs/rGO as an amplification scaffold for the improvement of the conductivity and electrocatalytic activity of the metallophalocyanines GoPc as shown in Figure 7. In this glucose sensor, a glassy carbon electrode was modified with the help of a single-walled carbon nanotube/reduced graphene oxide/cobalt-phthalocyanines nanohybrid (GCE-SWCNT/rGO/CoPc) for the non-enzymatic determination of human saliva glucose. The developed electrode showed promising potential for the development of a painless, non-invasive, and accurate glucose-sensing device.

#### 5.2.2. MWCNTs Based Nanosensors

**Figure 8:**
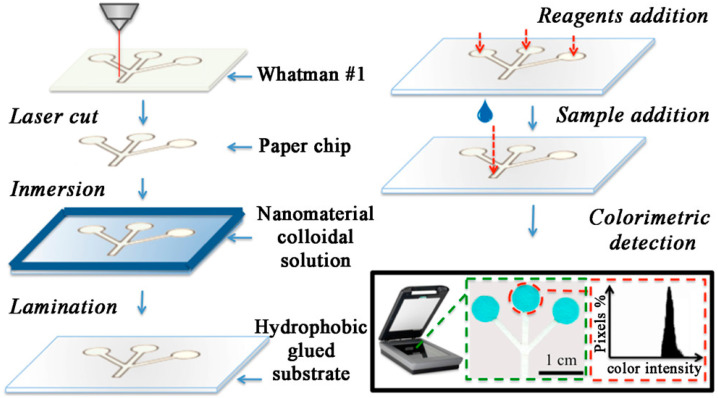
Schematic illustration of disposable microfluidic paper-based devices (μPADs). Image reproduced with permission from [183]. Copyright 2015 American Chemical Society.

Increasing the analytical performance of the sensors is one of the crucial requirements in the development of glucose sensors, and paper-based microfluidic devices combined with nanoparticles can be of particular interest. Disposable microfluidic paper-based devices (μPADs) are a promising new class of point-of-care systems. Paper-based analytical devices provide unique advantages for ultrasensitive sensing applications such as easy fabrication, affordable cost, and the ability to drive the flow without additional equipment. However, they need to add additives, which can interfere with the sensor performances by possible interaction with the enzyme activity (Figure 8). Figueredo et al. developed a microfluidic paper-based device with the help of Fe_3_O_4_ nanoparticles (MNPs), multi-walled carbon nanotubes (MWCNTs), and graphene oxide (GO), and the analytical performance of the device was studied with the help of bienzymatic colorimetric detection. To fabricate the device, a CO_2_ laser was used on 20 × 20 cm Whatman #1 filter paper. The laser was used to create the microfluidic channels and detection zones by cutting the paper. Microfluidic channels help to conduct the sample and the detection zone helps to create the color development. Three circular detection zones were created and interconnected by microfluidic channels. HP office scanner with 600 dpi was used to perform colorimetric measurements. This modified μPAD showed promising analytical performance allowing the low concentration visual detection of the glucose. The used modified POC device showed many advantages such as simple instrumentation, cost-effectiveness, and ease of operation. Combining the carbon and magnetic nanoparticles could offer greater potential in bioanalytical applications. 

In the case of electrochemical biosensors, enzymes play important roles to generate chemical signals by serving as biological recognition molecules. Enzymes show high thermal sensitivity and display catalytic activity only at an optimum temperature range. It is reported that enzyme-based biosensors could be temperature sensitive since they are dependent on strong temperature-dependent catalytic activity. It is important to develop sensors resistant to a wide temperature range. In this case, phase-changeable materials can be of particular interest due to their several advantages such as high-energy storage density, simple design, affordable cost, and great heat delivery capacity with controllable temperature. However, they cannot be used because of some drawbacks such as leakage, low thermal conductance, corrosiveness, and higher supercooling. Another study recently published by Sun et al. [184] developed an intelligent biosensor based on functionalized multi-walled carbon nanotubes (MWCNTs) along with phase change materials (PCMs). To overcome the drawbacks associated with PCMs, microencapsulation can be a promising strategy as reported in this study. This study demonstrated the thermal self-regulatory biosensor with the help of CNTs and PCMs for the detection of glucose sensing in a high-temperature environment. They developed a microcapsule system with PCMs as the core and CNT/SiO2 as the shell and used it to modify glassy carbon electrodes along with GOx. Such a system with the conjunction of GOx with the CNTs interface results in an effective conveyor belt to receive the electrons from the cofactor FAD (flavin adenine dinucleotide) in GOx.

### 5.3. Carbon Nanotube for Biological Components Detection

The detection of biological components is very important in biology, clinical science, and in hospitals facing real patients. In particular, it can make an early diagnosis of the disease, thereby allowing the patient to receive a faster and more correct response to the disease [185]. This is closely related to the quality of life of patients. However, even if the patient has a specific disease, if the biological component cannot be detected or not, the patient’s life will be very difficult and painful, the treatment cost will increase significantly, and a cure cannot be guaranteed. In fact, many patients around the world suffer from misdiagnosis and late diagnosis [186]. In order to fundamentally solve these problems, it is urgent and very important to develop materials, devices, and equipment that can detect biological components with high performance and high sensitivity.

**Figure 9:**
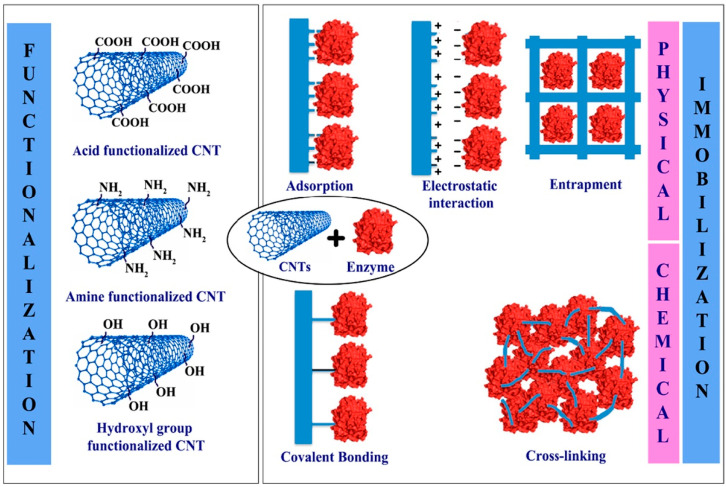
Schematic illustration of the functionalization of CNT and enzyme immobilization method. Image reproduced with permission from [169]. Copyright 2021 Elsevier.

CNTs have also been studied for use in the detection of biological components. CNTs have many advantages in the detection of various components due to their unique physical properties, such as large surface area [187], tubular three-dimensional structure [188], and the possibility of multiple modifications [189]. Here, we introduce studies of CNTs for the detection of biological components. Recently, much research has been conducted to develop a sensor that detects glucose using CNTs. The detection of glucose in the serum is very important to mankind and has been performed for a long time. Measurement of blood glucose levels, especially in diabetic patients, is indispensable that must be performed daily and frequently. Scientific, patient-friendly, and modern blood glucose level measurement began with Clinistix developed by Kohn in 1957 [190], and Dextrostix developed by Ernie Adams in 1965 [191]. In a recent glucose detection study, a biosensor using ZnFe_2_O_4_, CNT, and glucose oxidase was developed [151]. Briefly, ZnFe_2_O_4_ was conjugated with CNT by a one-step solvothermal approach using acid-treated CNT as a precursor. Glucose oxidase (GOD) was linked to ZnFe_2_O_4_-conjugated CNT by coupling reaction between the amine group and carboxyl group (ZnFe_2_O_4_-CNT-GOD). When glucose is added to ZnFe_2_O_4_-CNT-GOD, glucose is oxidized by GOD. In this process, the intermediate product, hydrogen peroxide, oxidizes the 3,3′,5,5′-tetramethylbenzidine (TMB) substrate and is eventually visualized in blue. In this process, ZnFe_2_O_4_ acts as a peroxidase and not only accelerates the overall reaction but also increases the intensity of the detected signal (Figure 9).

ZnFe_2_O_4_-CNT-GOD has a glucose detection range of 0.8 to 250 μM with a detection limit of 0.58 μM. ZnFe_2_O_4_-CNT-GOD did not react with lactose, maltose, fructose, sucrose, uric acid, dopamine, cystine, albumin, and ascorbic acid, so there was no component detection, but only a glucose-specific reaction occurred. In addition, the ZnFe_2_O_4_-CNT-GOD showed only a negligible change in the detection sensitivity of glucose even in the presence of 100 μM of copper, zinc, potassium, calcium, and iron ions. The authors report that the fabrication method of ZnFe_2_O_4_-CNT-GOD is simple, maintains the sensing activity for at least 20 days, and can be reused at least five times. Wang C et al. reported enzyme-functionalized CNTs and their application in glucose and Fe^2+^ detection [192]. Briefly, CNT was modified with carboxylation for functionalization. Later, carboxylated CNTs were covalently conjugated with GOD and/or horseradish peroxidase (HRP) (CNT-HRP-GOD). CNT-HRP-GOD detects glucose through the chain reaction between glucose, GOD, and HRP. In this process, the intermediate product, hydrogen peroxide, oxidizes the 3,3′,5,5′-tetramethylbenzidine (TMB) substrate and results in the production of colorimetric products (blue). Fe^2+^ reacts with hydrogen peroxide and leads to a lower concentration of hydrogen peroxide, which in turn decreases the oxidation state of TMB and eventually causes a lower colorimetric absorbance of the solution. CNT-HRP-GOD has a glucose and Fe^2+^ detection range of 1 to 100 μM with a detection limit of glucose and Fe^2+^ of 0.3 and 0.22 μM, respectively. CNT-HRP-GOD did not react with other sugars except glucose and did not react with albumin and ascorbic acid, so it was verified as a biosensor through a glucose-specific reaction.

Alcohol detection can be applied to a variety of fields, from the quality analysis of bio-alcohol, which has been actively researched and manufactured recently, to checking the blood alcohol concentration and analyzing the quality of alcoholic beverages. Wilson, T et al. reported a CNT-based alcohol biosensor [155]. They modified CNTs using polytyramine (PT) and glassy carbon (GC). PT has been electro-deposited onto MWCNT-modified GC electrodes via oxidation of tyramine (GC/MWCNT/PT). ADH immobilization for alcohol detection was improved by the PT layer. The polymeric film was formed on the electrode surface of MWCNT and it was confirmed using SEM and XPS. In order to detect alcohol with GC/MWCNT/PT, GC/MWCNT/PT was immersed in 0.05 M of sodium phosphate buffer (PBS) which contained 1 mg mL^−1^ of alcohol dehydrogenase (ADH). The EDC/NHS coupling reaction was performed for one hour for the conjugation of the two substances (GC/MWCNT/PT/ADH). This alcohol biosensor showed a sensitivity of 4.28 ± 0.06 μA mM^−1^  cm^−2^, a regression coefficient of 0.9993, and a response time of 5 s. Furthermore, it had a 10 μM limit of detection and it almost accurately detected the alcohol content of commercial alcoholic beverages at a level of recovery of 97.4–102.1%.

Ascorbic acid is one of the water-soluble vitamins, and since L-gulono-γ-lactone oxidase is not present in the body, ascorbic acid must be consumed as a food or nutritional supplement [193]. A deficiency of this causes scurvy [194]. Ascorbic acid acts as an antioxidant in the body, protecting normal cells from various reactive oxygen species [195] and helping to maintain the immune system [196]. In addition, it suppresses various inflammatory reactions [197] and aging [198], and helps the elderly to maintain cognitive ability and memory, thereby helping to prevent Alzheimer’s disease [199]. Zhao, Y et al. reported an ultra-sensitive biosensor for the voltammetric determination of ascorbic acid (AA) [200]. For the fabrication of CNT-based high-sensitivity ascorbic acid sensors, MWCNTs were surface modified with glassy carbon electrodes (GCEs), graphene oxide (GO), and gold nanorods (AuNRs). Since the aggregation of MWCNTs reduces the detection sensitivity of biological components, prevention of aggregation improves the sensitivity. In this study, they used GO to prevent the aggregation of MWCNTs. Furthermore, overpotential was reduced and the peak current of AA oxidation was increased by positively charged AuNRs. The electrochemical properties of biosensors were investigated by cyclic voltammetry (CV). Finally, this ascorbic acid biosensor sensitively detected ascorbic acid with low working potential (0.036 V), low detection limit (0.85 nM), and high sensitivity (7.61 μA μM^−1^ cm^−2^).

**Figure 10:**
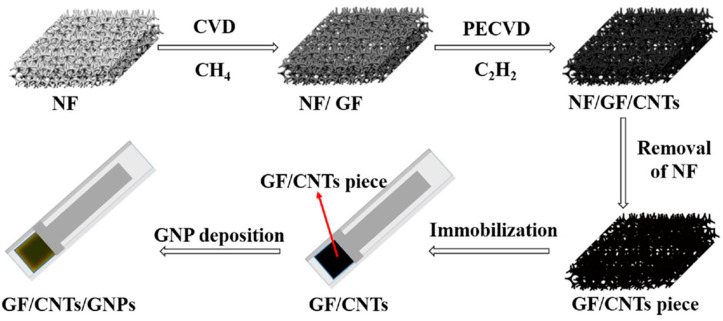
Schematic illustration of GF/CNTs/GNPs preparation for uric acid detection biosensor. Image reproduced with permission from [207]. Copyright 2017 Elsevier.

CNTs can be applied as biosensors for the detection of uric acid and it has already been proven in several studies [201,202,203]. Some chronically ill patients have hyperuricemia, in which there is too much uric acid in the body [204]. It accumulates in the cartilages and produces tophi and uric acid crystals, which cause great pain to the patient. In addition, the accumulation of tophi and uric acid induces an inflammatory response in the cartilages and causes lasting damage to cartilage and bones [205]. This phenomenon also entails great pain for the patient. Tophi and uric acid accumulate in the kidneys as well as in the cartilages, and just as they accumulate in the cartilages, they cause very severe organ irritation and pain [206]. For the detection of uric acid, Huang B et al. reported a standing electrochemical sensor based on CNT for the determination of uric acid [207]. They developed a free-standing electrochemical biosensor. CNT was modified with 3D graphene foam (GF) and gold nanoparticles (GNPs) for the fabrication of biosensors (GF/CNTs/GNPs). Cyclic voltammetry (CV) and differential pulse voltammetry (DPV) were used for the investigation of the electrochemical properties of GF/CNTs/GNPs. GF/CNTs/GNPs show outstanding electrocatalytic activity toward dopamine and uric acid. Detection of uric acid with GF/CNTs/GNPs shows remarkable sensitivity of 3.36 μA μM^−1^ cm^−2^, the low detection limits of 33.03 nM (S/N = 3), with a wide linear range of 0.50–60 μM. Furthermore, GF/CNTs/GNPs evaluated the quantification of uric acid with human urine. GF/CNTs/GNPs show good agreement in the concentration of uric acid (μM), total found (μM), and recovery (%) with high-performance liquid chromatography (Figure 10).

CNT-based biosensors can detect not only single molecules but also protein-based components. Here, we introduce the detection of biological components for the diagnosis of Alzheimer’s disease. Amyloid-β accumulates in the brains of people with Alzheimer’s disease and this phenomenon is a pathological mechanism of Alzheimer’s disease [208]. Amyloid precursor protein is one of the proteins that plays a very important role in regulating the homeostasis of the neuronal system, such as neuronal development and signal transfer between neurons [209]. However, various precursor protein cleavage products produced by the cleavage of amyloid precursor protein are very closely related to Alzheimer’s disease and induce dysfunction of the neuronal system [210]. Oh, J et al. reported a carbon nanotube (CNT) film-based biosensor with a metal-semiconductor field effect transistor structure (MESFET) for amyloid-β detection in human serum [211]. Briefly, for the fabrication of CNT-MESFET, the top gate was modified by depositing Au (10 nm) only in the middle of the semiconducting CNT channel. These immobilized antibodies on CNT-MESFETs were controlled by the antibody-binding proteins. In order to detect HRP used as the model analyte, anti-HRP antibodies were immobilized on the Au top gate with protein G or auto-displayed Zdomains of protein A as the antibody-binding protein. CNT-MESFET exhibited a higher sensitivity than the antibodies immobilized biosensor using a chemical linker. CNT-MESFET could detect the HRP at levels as low as 1 fg mL^−1^ in serum. Finally, they applied the CNT-MESFET to the detection of amyloid-β in human serum. This CNT-MESFET could detect the amyloid-β at the level of 1 pg mL^−1^ in human serum. It can be applied as a CNT-based biological material detection sensor with very high sensitivity, and further research is needed to see if it can detect other substances besides amyloid-β.

Thrombin is a protein closely related to blood clotting [212]. During bleeding, platelets are destroyed, and thromboplastin is released into the plasma, which is activated in the presence of calcium ions in the blood and becomes thrombin. It catalyzes the reaction of hydrolysis of soluble fibrinogen in the blood, which is the essence of blood coagulation, into insoluble fibrin [213]. Therefore, quantitative detection of thrombin in bleeding patients or patients undergoing surgery can prevent accidents caused by bleeding and judge the patient’s condition for bleeding more clearly. Su, Z et al. reported an amperometric thrombin aptamer sensor (aptasensor) as a thrombin biosensor [165]. For the fabrication of the aptasensor, polyaniline-coated MWCNT was placed on the glassy carbon electrode (GCE). Later thiolated thrombin-specific aptamers were conjugated with polyaniline by the thiol-ene reaction. The surface of the aptasensor was coated with bovine serum albumin to prevent non-specific binding. The modified GCE shows a pair of well-defined redox peaks (at 50/−25 mV) and the tethered TTA–thrombin interaction shows a decreased electrochemical signal. Thrombin in spiked human serum (0.2 to 4 nM) was accurately detected by the aaptasensor and it shows recoveries that ranged from 95 to 102%.

**Figure 11:**
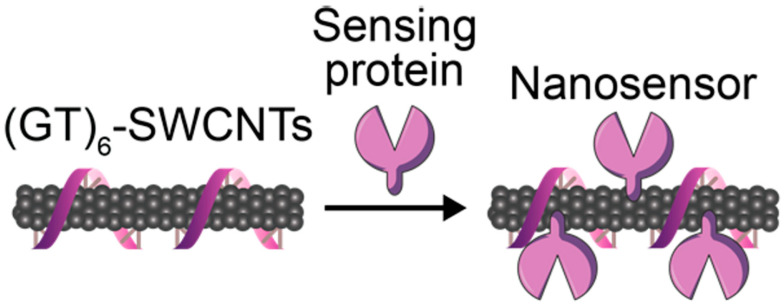
Schematic representation of fabrication of ACE2-(GT)6-SWCNTs nanosensor using ACE2 as a sensing protein. Image reproduced with permission from [179]. Copyright 2021 American Chemical Society.

Accurately detecting COVID-19 is very important in the situation of the pandemic. Early detection and accurate diagnosis of the virus can prevent the spread of coronavirus infection. In addition, diagnosis of severe acute respiratory syndrome coronavirus 2 (SARS-CoV-2) is crucial for tracking the route of transmission and suitable treatment for patients in the event of a pandemic [214,215]. Pinals, R et al. introduced an SWCNT-based optical sensing approach toward this end [179]. SARS-CoV-2 enters the host cell through binding to the ACE2 receptor [216]. They used ACE2 to fabricate the noncovalently functionalized SWCNT as a virus sensor since ACE2 has a high binding affinity to the SARS-CoV-2 spike protein. Biosensor fluorescence was increased (2-fold) in the presence of the SARS-CoV-2 spike protein. They evaluated biosensor stability and confirmed preserving sensing responses in saliva and virus delivery media. In addition, it was demonstrated that the biosensor had a 73% fluorescence-on response within 5 s of exposure to 35 mg L^−1^ SARS-CoV-2 virus-like particles. The biosensor shows a 100% turn-on response in fluorescence upon the addition of 1 μM CoV-2 S spike protein receptor-binding domain (S RBD) (Figure 11).

**Figure 12:**
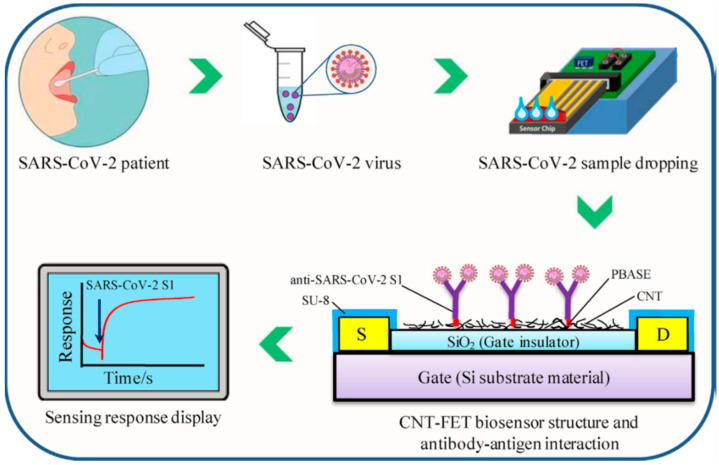
Schematic illustration of SARS-CoV-2 S1 testing steps of CNT-FET biosensor. Anti-SARS-CoV-2 S1 was conjugated on the CNT (using PBASE as a linker) to produce SARS-CoV-2 S1 detectable CNT-FET biosensor. Image reproduced with permission from [28]. Copyright 2021 Elsevier.

Zamzami, M et al. developed a fast (2–3 min), easy-to-use, low-cost, and quantitative electrochemical biosensor based on a CNT field-effect transistor (CNT-FET) that allows digital detection of the SARS-CoV-2 S1 [28]. It can quickly and accurately detect SARS-CoV-2 S1 antigens in saliva samples. The anti-SARS-CoV-2 S1 was immobilized on a Si/SiO2 surface by CNT printing for the fabrication of a CNT-FET biosensor. The CNT-FET biosensor effectively detected the SARS-CoV-2 S1 antigen in 10 mM ammonium acetate buffer at concentrations from 0.1 fg mL^−1^ to 5.0 pg mL^−1^. The limit of detection (LOD) of the CNT-FET biosensor was 4.12 fg mL^−1^. In order to confirm whether the biosensor can specifically detect only the target antigen, selectivity tests were performed using target SARS-CoV-2 S1 and non-target SARS-CoV-1 S1 and MERS-CoV S1 antigens. The biosensor has good detection sensitivity with SARS-CoV-2 S1 antigen. However, it shows no detection response to SARS-CoV-1 S1 and MERS-CoV S1 antigen. The developed CNT-FET biosensor was verified to be capable of sensitive, fast, and accurate detection of SARS-CoV-2 S1 in human saliva (Figure 12).

### 5.4. Carbon Nanotube for Bacteria and Virus Detection

**Figure 13:**
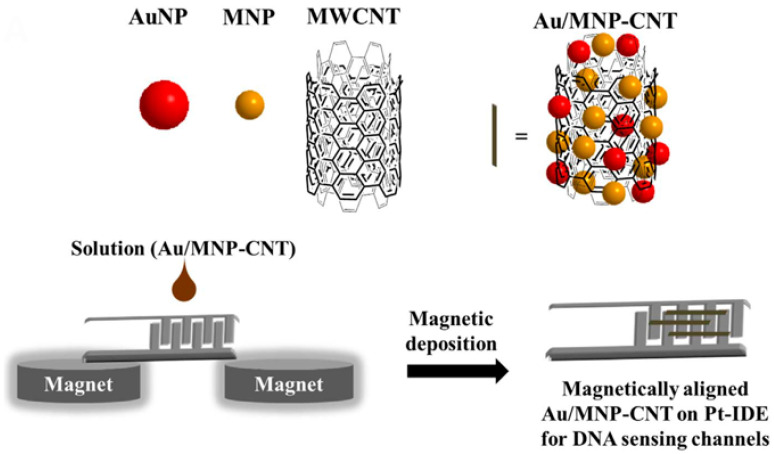
Schematic illustration of fabrication of magnetically aligned gold/magnetic nanoparticles decorated CNT on Pt-IDE for virus DNA-sensing platform. Image reproduced with permission from [219]. Copyright 2018 Elsevier.

As mentioned before, CNTs and their derived structures have excellent physical properties including electrical conductivity, SERS, FRET, and so on. They can be utilized as sensing channels to detect bacteria, viruses, virus DNA, etc. [217,218]. Especially, the need for high-performance virus sensing platforms has increased for well-being and better human life, so many CNT-based sensing systems have been introduced. For example, Lee and his co-workers introduced virus DNA detection via an electrical biosensing platform which is composed of magnetically aligned NPs decorated CNT on the IDE (Figure 13) [219]. In this study, firstly gold and magnetic nanoparticles were modified on the surface of CNT (Au/MNP-CNT) and they were laid on the Pt-IDE via an external magnetic field. After that, the thiol-modified probe DNA was immobilized on the Au NP. In this case, Au and CNT played a role as electrical sensing channels and MNP was the moiety for alignment. Due to the synergic properties between the three nanomaterials, this sensing platform showed high sensitivity with a LOD of 8.4 pM for influenza virus DNA and 8.8 pM for norovirus DNA. Furthermore, it showed high selectivity against mismatched DNA strains. Therefore, this sensing platform could show excellent sensing performance. 

On the other hand, metallic nanoparticles (NPs)-decorated CNT was also used as a plasmonic substrate for a plasmonic resonance energy transfer (PRET)-based FL immune sensing system. In this case, metallic NPs such as gold or silver NPs and CNT possess plasmonic properties; thus, their hybrid structure-based plasmonic material has synergic properties. For instance, the plasmonic property of gold NP-decorated CNT (gold-CNT) assisted in the detection of influenza viruses [220]. In this study, gold-CNT was modified with influenza virus Ab, so it could capture the target virus and subsequently, fluorescent quantum dots (QDs)-Ab were added into the mixture and bound with virus-Ab-gold CNTs, and finally, sandwich structures were induced. As a result, depending on the concentrations of the target influenza virus, the FL of QD was changed linearly. According to the results of detection performance, the limit of detection of viruses was estimated at around 0.1 pg mL^−1^. Furthermore, the influenza virus from a clinical sample was also monitored with excellent sensitivity was 50 PFU mL^−1^ in the range of 50–10,000 PFU mL^−1^. It meant that a metal NP-CNT structure-based fluoro-immuno sensing system could be potentially applied for virus detection. 

In another study, CNT could be utilized as a sensing channel to detect the dengue virus (DENV) via an electrochemical approach. Wasik et al. fabricated a heparin-SWCNT hybrid structure on the electrode to monitor the dengue virus and the resistance difference was measured depending on the concentration of the virus [221]. The limit of detection of this system showed 8 DENV/chip and this system showed excellent selectivity against the influenza virus. Therefore, a CNT-based electrochemical sensing platform also could be developed for high-performance biosensors.

On the other hand, some bacteria have caused critical diseases, and they have threatened human life. So, a highly sensitive and selective bacteria sensing system was required, and several bacteria were also successfully detected by using a CNT-based sensing platform. For instance, Zhang et al. reported a gold-CNT-based sensing system that could detect *Escherichia coli* (*E. coli*) through an electrochemical approach [222]. Firstly, they fabricated the gold-CNT/GCE for the sensing channel. Then, the captured Ab was immobilized on the surface of gold-CNT to monitor the *E. coli*. The sensing behavior of the gold-CNT-based sensing system was characterized depending on the amounts of bacteria from 2.0 × 10^2^ to 2.0 × 10^6^ CFU mL^−1^. This system showed linear response depending on the amount of *E. coli* and good sensitivity and selectivity was also proven. Especially, *E. coli* in sludge was also successfully detected, thus this system exhibited excellent sensing performance. 

In another study, the detection of *E. coli* O157:H7 based on an MWCNT electrical sensing system was introduced by Li and co-workers [223]. In this study, MWCNT was covered by polystyrene sulfonate (PSS) and the authors produced a PSS-MWCNT-based layer-by-layer (LBL) structure using poly(ethyleneimine) (PEI) to apply for the electrical sensing channel. O157:H7 Abs were modified on the surface of MWCNT to capture the target bacteria. The electrical signal was monitored depending on the concentration of O157:H7 and a linear response was shown. Interestingly, the authors collected the bacteria that were captured (O157:H7) by the sensing channel and isolated specific DNA from the collected O157:H7. Subsequently, the concentration of O157:H7 DNA was estimated by loop-mediated isothermal amplification (LAMP). Based on the sensing performance results, the LOD of this system was around 1 PFU mL^−1^. Therefore, they developed highly sensitive bacteria detection systems using MWCNT.

*E. coli* in dairy products were also detected by using CNT and gold NP mixture [224]. In this case, the surface of CNT was modified with target Ab by an EDC/NHS coupling reaction and horseradish peroxidase (HRP) enzyme at the same time. On the other hand, gold NPs were coated by poly(amidoamine) dendrimer and specific Ab for *E. coli* was attached to the surface of NPs. In this environment, if target bacteria existed in the sample, these two nanomaterials could be formed as a sandwich structure with bacteria and its electrochemical sensing signal might be changed. The sensing performance of this system was tested under a range of 1.0 × 10^2^ to 1.0 × 10^6^ CFU mL^−1^ and a LOD of around 50 CFU mL^−1^ was estimated. Therefore, a CNT-based sensing system could be applied to the bacteria screening platform.

Liu et al. have reported that molecular imprinted TiO_2_-coated multiwalled carbon nanotubes (MI-TiO_2_@CNTs) were fabricated to detect microcystin-LR (MC-LR), a type of cyanobacterial toxin in water by the photoelectrochemical method. The molecular imprinted TiO_2_ showed enhanced detection in comparison to traditional TiO_2_ and non-imprinted TiO_2_. This sensor resulted in a wide linear range from 1.0 pM to 3.0 nM for the detection of MC-LR. MI-TiO_2_@CNTs achieved magnificent selectivity towards MC-LR. Moreover, this promising sensor showed high sensitivity for the detection of MC-LR which could be a potential candidate for water purification [29].

He and co-workers developed an enzyme-free and dual-signal readout immunosensor that was used to detect MC-LR while an enzyme-based biosensor with great obstacles such as instability, sensitivity, temperature, and pH should be considered. Initially, gold nanoparticle-decorated CNT (AgNP-CNTs) was fabricated for the detection of MC-LR and secondly, silver nanorods were coated over AgNP-CNTs to detect via dual-signal mode. These sensors showed the determination of MC-LR in a linear range from 0.005 μg L^−1^ to 20 μg L^−1^ with a LOD of 2.8 ng L^−1^. In terms of reproducibility, high selectivity, and sensitivity, these sensors indicated their promising application in environment monitoring [225].

In another study, Han et al. proposed an MWCNT-based electrochemical biosensor that was demonstrated to monitor the MC-LR in drinking water supplies. This biosensor was fabricated in well-aligned and millimeter-long MWCNT arrays by water-assisted CVD. In addition, monoclonal antibodies were decorated to specify MC-LR toxin detection. A linear range from 0.05 to 20 μg L^−1^ was observed for the detection of MC-LR with a LOD of 1 μg L^−1^ in drinking water [226]. To reduce the burden of cost-effectiveness and increase the rapid detection of MC-LR in environmental water, Queiros and co-workers proposed label-free potentiometric sensors composed of MWCNTs. These sensors were synthesized by imprinted polymer and polyvinyl chloride membranes. This method was applied successfully to detect MC-LR with great selectivity and sensitivity. Moreover, this method benefited with easy production and cost-effectiveness [227].

Cholera is another devastating disease that has taken uncountable lives over the past few decades. The detection of cholera toxin (CT) was highly required to eradicate cholera from our lives. Viswanathan et al. proposed a sensitive method to detect CT by using an electrochemical immunosensor. This immunosensor was composed of potassium ferrocyanide, ganglioside (GM1)-functionalized liposomes, and monoclonal antibodies on the surface of Nafion-supported multi-walled carbon nanotubes. The detection mechanism was proposed by a sandwich-type assay, where the toxin was first coupled with an anti-CT antibody and followed by a GM1-functionalized liposome. This sandwich method resulted in the detection of CT in ultra-trace levels. The detection of CT showed a linear range of 10^−14^−10^−7^ g mL^−1^ with a LOD of 10^−16^ g of CT [228].

In another study, Palomar et al. proposed an impedimetric immunosensor based on CNTs to improve sensing performances by increasing electroactive surface areas on CNTs. These systems were modified with polypyrrole-nitrilotriacetic acid (poly(pyrrole-NTA)) and Cu (II) complex to produce sensor devices. With great sensitivity and easy reproducibility, the cholera sensor showed a promising linear detection range from 10^−13^–10^−5^ g mL^−1^ with a LOD of 10^−13^ g mL^−1^, which could be a potential sensing platform to detect cholera in the environment [229].

Additionally, we have made efforts on CNT-based biosensors on the action of detection methods (Table 3).

## 6. Commercialization of Carbon Nanotube as Sensors

Despite great achievements in CNT-based biosensors, their commercialization is still under investigation. Over recent years, many efforts have been explored to fabricate devices with CNTs and utilize them to interact with analytes in controlled ways. The mechanism of sensing and interaction between CNTs and analytes is complicated and actively debated for generalization. To avoid non-specific binding (NSB) on CNTs rather than the analytes of interest, sophisticated methods are being demanded. Some attempts such as using blocking agents have been investigated to minimize the NSB molecules to CNTs and sensing activities. Ye et al. reported that using the lipid bilayers can influence the sensing of transmembrane and signaling phenomena. These methods have gained great attention to suppress NSB. 

Many challenges have arisen towards device fabrication due to having controlled synthesis of CNTs, and limits for commercialization. Interestingly, some exciting results showed a hope to produce CNTs in terms of reasonable size and shape. Moreover, the production of high-performance devices requires a high density of well-aligned CNTs as the backbone of transportation, while the chirality of CNTs leads to differing device characteristics. Therefore, many strategies such as surface modification and electrical burn-off of CNTs have been applied to shape more homogeneity. One of the promising strategies was functionalization on the surface of CNTs, which has been explored to differentiate and sort them, which raised another obstacle in terms of cost-effectiveness and separation techniques [266]. Sophisticated and outstanding development in sciences has enriched the technology over the years which has resulted in a cost reduction in device production. Interestingly, reliable sensor response has been investigated to specific analytes by fabricating CNTs [267,268]. Furthermore, several outcomes are successfully integrated with CNTs for commercial devices.

Therefore, for the production and release of new cost-effective electronics onto the market, research on prolific CNT synthesis processes would be of foremost relevance. The exploration of CNT toxicity would be one of the obstacles to be surmounted prior to the large-scale development of CNTs as new sophisticated functional materials in the industry. Since these materials are both novel and microscopic, this challenge is accentuated; the assessment of a material’s toxicity is made more onerous due to the tendency of materials to exhibit unique behaviors in the nanoscale range as a result of quantum phenomena. Reviews of the toxicological implications of CNTs are conflicting. According to a pilot study, when CNTs are injected into the abdominal cavity of mice, within a week they exhibit asbestos-like pathogenicity on their contact with the lungs, causing granulomas to develop on the lungs’ mesothelium cells. The toxicity in CNT (thinner than 3 nm and longer than 20 nm) [269] induces due to their elongated, fiber-like morphology that mimics asbestos and is governed by the length of the CNTs [270]. However, some studies that contradict these results claim that CNTs only have a small toxicological impact on humans. The interaction of SWCNTs with different human cells, especially lung cells, has been found to exhibit no changes in cellular viability [271]. However, deep and accurate interaction between CNTs and human cells is required to get practical output in terms of commercialization of CNTs as biosensors, especially in vivo employment. The challenges associated with realistic applications of CNTs-based sensors towards integration with analytical devices and fabrication on an industrial scale. Impurities and defects of CNTs are to be concerned cautiously as they hamper the original properties of CNTs for their applications. Importantly, the purification techniques are crucial for large-scale production to translate into commercialization [272]. Interestingly, the recent advancement in device fabrication with CNTs has been upgraded as disposable, even single isolated CNTs have also been integrated into commercialization.

## 7. Conclusions

Over the years, researchers and scientists have used a diverse range of nanomaterials such as metal nanoparticles (NPs), metal oxide NPs, nanofibers [273], quantum dots (QDs), and carbon nanomaterials such as carbon quantum dots [274], graphene, and carbon nanotubes (CNTs) to fabricate high-performance and sensitive biosensors. CNTs and their derivatives have gained great attention in the field of advanced functional materials today. It has been explored in diverse fields from defense to electronics. The field of biomedical applications has investigated CNTs and their derivatives extensively as potential candidates. Although the physical and chemical properties are not completely understood, it has been exploited by the electronics industry over the years. CNTs showed excellent properties in device fabrications as well as sensing behaviors. CNTs and their derivatives have been utilized for bio and chemical sensing due to having similar sizes to the analytes and bio-species. Due to their small size and high aspect ratio, CNTs exhibit unusual optical, mechanical, electrical, and chemical properties due to their small diameter and high aspect ratio. Utilizing them, a wide class of sensors is fabricated. It has been shown that CNTs have improved cell penetration properties and stability, as well as chirality and diameter-based physicochemical properties. On the account of synthesis, the materials that are necessary for CNT production are profuse, and they can be crafted with only a modest amount of raw materials. Further functionalization without damaging the covalent backbone extends the desired application of CNTs. Although one major drawback of CNT production is reproducibility, structurally and chemically reproducible batch production with minimal impurities is an immediate concern. Another two important properties that distinguish them from other nanomaterials are temperature stability (2800 °C in vacuum and ~750 °C in air) and hydrophilicity. While considering the mechanical properties, extremely high Young’s modulus values (1–1.8 TPa range) allow them to act as an excellent candidate for probe tips for scanning microscopy. Although some other disadvantages are always associated with CNTs, namely cellular toxicity, incompatibility with biological mediums, agglomeration, accumulation, and long-term persistence which require a strong action for mitigation. Numerous studies on CNTs and their derivatives have reported their interactions with analytes and their toxicology profiles. However, to allow their commercialization, there are limits in terms of cost-effectiveness, purity, and high density of perfect alignment during industrialization. A huge number of studies are conducted on biosensors to enable their commercialization. Interestingly, CNTs have been investigated as biosensors as in vivo devices, while many efforts have been made to minimize their toxicity profile. 

## 8. Future Perspective

CNTs are one of the most exciting and intriguing materials to the materials sciences and sensing platforms, particularly biomedical applications. Reducing the burden of synthesis of CNT-based biosensors, researchers are focusing to avoid the utilization of enzymes while increasing interest in the fabrication of other materials for CNTs [275]. Some studies have reported that the combination of CNTs and metals such as Au, Pt, etc., resulted in a great sensitivity and enhanced LOD. Another concern is how to make thermal stability and enhance the lifetime of CNT-based biosensors. Moreover, molecular modeling must be implemented along with experimental investigation in the development of promising CNT-based biosensors.

The research on CNTs is being redoubled after the coronavirus pandemic in health services as well as sensing industries. With their great properties, CNTs have found themselves as potential candidates to overcome their challenges and impact on aspects of health care as well as the environment.

## Figures and Tables

**Table 1:** Summarization of CNTs and their derivatives as biosensors for cancer detection.

Functionalization of CNTs	Sizes	Diagnosis Methods	Type of Cancer	Ref.
PEG-conjugated SWCNT functionalized with anti-CD44 antibody	1–2/200 nm	MRI/SPECT/NIR fluorescence imaging with nanoprobe	Breast cancer stem cells by monitoring iron content.	[134]
High-purity CNT films with gold nanoparticles (AuNPs)	Thin film layer	Field-effect transistor (FET) biosensor	Breast cancer based on detection of exosomal miRNA (miR-FET)	[141]
CNT chip coated with thin film with anti-EpCAM antibody	CNT film (10 mm × 10 mm)	pH responsive CNT film based microfluidic device capturing EpCAM expressing CTC	Various EpCAM-expressing circulating tumor cells (CTCs)	[135]
Functionalized multi-walled carbon nanotubes (MWCNTs) with anti-CA19-9 antibody	-	PEI-CNT film immobilized with antibodies (anti-CA19-9) was studied using the PM-IRRAS technique	Colon cancer cells (HT-29) expressing CA19-9	[137]
Hyaluronic acid (HA)-conjugated MWCNTs with anti-CD44 antibody	ITO substrate with standard size [7.5 (width) × 25 (height) mm]	Ligand–protein recognition [hyaluronic acid (HA)-CD44] assay. Electrochemistry can convert target analytes into the signal output.	Lung and breast cancer cells expressing CD44	[142]
Nanocomposite containing polyaniline (PANI) and CNT with anti-VEGF RNA aptamer on tumor cells.	CNTs diameter 1.5 nm and length = 1–5 µm)	VEGF detection by electrochemical sensor using RNA aptamer on screen-printed carbon electrode (SPCE)	VEGF-expressing cancer cells	[139]
Photo-luminescent SWCNTs engineered to respond metastatic prostate cancer biomarker (uPA).	-	Act by modulating the optical bandgap upon interaction with analyte	Metastatic prostate cancer biomarker (uPA)-expressing cell line	[143]
DNA/RGD-peptide/SWCNTS as nIR labels	Length 350 nm	ssDNA-peptide non-covalently adsorbed on SWCNTs and recognize cell surface receptors like integrins.	Integrin receptor-expressing cancer cells	[144]
DA-coated MUC1-functionalized MWCNTs	MWCNTs (diameter: 4–5 nm, length: 0.5–1.5 μm)	Electrochemical immunosensor based on dopamine coated MUC1 functionalized multi-walled carbon nanotubes sensing MUC1 biomarker	MUC1-expressing cancer cells	[140]
Enzyme aggregate-conjugated CNTs.	-	Emitted chemiluminescence by nanosensor catalyzed substrates will be detected in different time windows	Hepatocellular carcinoma	[145]

**Table 2:** Summarization of CNT-based enzymatic and non-enzymatic biosensors.

Methods	Analytes	Limit of Detection	Detection Range	Ref.
**Enzymatic**	Lactate	Not reported	5–20 nM	[149]
	Uric acid	9.91 μM	50 to 650 μM	[150]
	Glucose	0.58 μM	0.8 to 250 μM	[151]
	Glucose	3 × 10^−4^ M	(1–15) × 10^−3^ M	[152]
	Glucose	5 × 10^−5^ M	(0–5) × 10^−3^ M	[153]
	Glucose	2.99 × 10^−6^ M	(3–14) × 10^−3^ M	[154]
	Ethanol	1 × 10^−5^ M	(1–5) × 10^−4^ M	[155]
	Urease	67 μM	1.0–25.0 mM	[156]
	Alcohol dehydrogenase	10 μM	0.1 to 0.5 μM	[152]
	Choline	0.6 μM	3–120 μM	[157]
**Non-enzymatic**	Pyruvic acid	0.048 μM	0.1–200 μM	[158]
	Human epidermal growth factor receptor 2	7400 pg/mL	10–110 ng mL^−1^	[159]
	Cholesterol	0.5 nM	0.001–3 μM	[160]
	glucose	500 nM	2–19,600 μM	[161]
	Zearalenone	0.15 pg mL^−1^	0.001–0.1	[162]
	Long non-coding RNAs	42.8 fM	10^−14^–10^−7^ M	[163]
	MicroRNA 21	0.01 fM	10^−17^–10^−6^ M	[164]
	Thrombin	0.08 pM	0.001–4 nM	[165]
	Human epidermal growth factor receptor 2	50 fg mL^−1^	0.1 pg mL^−1^–1 ng mL^−1^	[166]
	Cardiac troponin T	0.04 pg mL^−1^	0.1–8 pg mL^−1^	[167]
	Urea	4.7 nM	0.066–20,600 µM	[168]
	Ascorbic acid	0.85 nM	0.001–8000 µM	[169]
	Glucose	645 nM	20–10,500 µM	[170]
	Glucose	0.33 nM	10–2000 µM	[171]
	Dopamine	9.5 nM	0.033–1 µM	[172]
	Potassium ions	Not reported	1000–32,000 µM	[173]
	Hydrogen peroxide	Not Reported	5 × 10^−6^–5 × 10^−3^ M	[174]
	MicroRNA 155	3.34 × 10^−14^ M	1 × 10^−13^–1 × 10^−9^ M	[175]
	Digoxin	7.95 × 10^−12^ M	2.65 × 10^−11^–6.8 × 10^−10^ M	[176]
	Sequence specific to chronic myelogenous leukemia	1 fM	10^−15^–10^−6^ M	[177]
	Myeloperoxidase	327 ng mL^−1^	Not reported	[178]
	SARS-CoV-2 spike protein	35 mg L^−1^	Not reported	[179]
	SARS-CoV-2 spike protein	0.55 fg mL^−1^	0.0055–5.5 pg mL^−1^	[180]
	Ascorbic acid	76.5 pM	100 pM to 1 mM	[181]

**Table 3:** CNT-based biosensors on action of detection methods.

Methods	Analytes	Limit of Detection	Detection Range	Ref.
Amperometric	Zearalenone	0.15 pg mL^−1^	0.001–0.1 ng mL^−1^	[162]
	Polyclonal anti-*Staphylococcus aureus*	100 CFU mL^−1^	10^2^–10^5^ CFU mL^−1^	[230]
	Glucose	645 nM	20–10,500 µM	[170]
	Glucose	0.33 nM	10–2000 µM	[171]
	Glucose	500 nM	1–1000 µM	[231]
	Glucose	3 × 10^−4^ M	(1–15) × 10^−5^ M	[152]
	Glucose	5 × 10^−5^ M	(0–5) × 10^−3^ M	[153]
	Glucose	2.99 × 10^−6^ M	(3–14) × 10^−3^ M	[154]
	Glucose	5 μM	8 μM–1.5 mM	[232]
	Alcohols	3.3 × 10^−3^ M	(12.5–100) × 10^−3^ M	[152]
	Ethanol	1 × 10^−5^ M	(1–5) × 10^−4^ M	[155]
	Xanthine	1.2 × 10^−7^ M	(2–86) × 10^−6^ M	[233]
	Choline	6 × 10^−7^ M	(3–120) × 10^−6^ M	[157]
	Nitrite	0.4 μM	1–1000 μM	[234]
	Urine albumin	4.96 × 10^−8^ mol L^−1^	3.3 ng μL^−1^–3.3 mg μL^−1^	[235]
	Cholesterol	0.1 × 10^−3^	2–8 × 10^−3^ M	[236]
Fluorescence	Adenosine triphosphate	2.4 × 10^−7^ M	Not Reported	[237]
	Troponin T	2.5 × 10^−9^ M	Not Reported	[238]
	Acetic acid	0.05% (*v*/*v*)	0.05–3.2% (*v*/*v*)	[239]
	SARS-CoV-2 spike protein	35 mg L^−1^	Not reported	[179]
	Hydrogen peroxide	Not Reported	5 × 10^−6^–5 × 10^−3^ M	[174]
	MicroRNA 155	3.34 × 10^−14^ M	1 × 10^−13^–1 × 10^−9^ M	[175]
	Digoxin	7.95 × 10^−12^ M	2.65 × 10^−11^–6.8 × 10^−10^ M	[176]
Chemiresistive	*E. coli* O157:H7	10^5^ colony-forming units (CFU) mL^−1^ (whole cell); 10^3^ CFU mL^−1^ (lysates)	10^3^–10^7^ CFU mL^−1^	[240]
	Cardiac myoglobin	1 ng mL^−1^	1–1000 ng mL^−1^	[241]
	histidine rich protein II (HRP2)	0.97 fg mL^−1^	10 fg mL^−1^–10 ng mL^−1^	[242]
	Avian influenza virus (H5N1) DNA sequence	Not Reported	2–200 pM	[243]
	Microcystin-LR	0.6 pg mL^−1^	0.001–1 ng mL^−1^	[244]
Differential pulse voltammetry (DPV)	Bisphenol A	4 nM	0.01–0.7 µM	[245]
	Ascorbic acid	0.85 nM	0.001–8000 µM	[169]
	Dopamine	1.36 nM	0.1–48 µM	[207]
	Dopamine	0.87 nM	0.005–100.0 μM	[246]
	Uric acid	33.03 nM	0.5–60 µM	[207]
	Anthrax lethal factor	3.5 fM	10^−14^–10^−10^ M	[247]
	Hepatitis B virus genomic DNA	2.5 fM	10^−14^–10^−8^ M	[248]
	Folic acid (vitamin B_9_)	0.095 μmol L^−1^	0.5–26 μmol L^−1^	[249]
	Matrix metalloproteinase-7 (MMP-7)	6 pg mL^−1^	1 × 10^−2^–1 × 10^3^ ng mL^−1^	[250]
	Daunorubicin Tamoxifen	3.0 nM 0.1 μM	0.008–350 μM 0.5–330 μM	[251]
	Long non-coding RNAs	42.8 fM	10^−14^–10^−7^ M	[163]
	Sequence specific to *E. coli*	17 × 10^6^ fM	Not reported	[252]
	MicroRNA 21	0.01 fM	10^−17^–10^−6^ M	[164]
	Thrombin	0.08 pM	0.001–4 nM	[165]
	Methotrexate	70 nM	0.7–100 µM	[253]
	Staphylococcus aureus	15 CFU mL^−1^	10–10^7^ CFU mL^−1^	[254]
Cyclic voltammetry (CV)	Methotrexate	70 nM	0.7–100 µM	[253]
	Urea	4.7 nM	0.066–20,600 µM	[168]
	3-ocatnone	0.3 ppb	0–0.0025% (*v*/*v*)	[255]
	Butanone	0.5 ppb	0–0.055% (*v*/*v*)	[255]
	Carcinoembryonic antigen	8.39 pg mL^−1^	10 pg mL^−1^ to 10 ng mL^−1^	[256]
	Semicarbazide	0.025 ng mL^−1^	0.04–7.6 ng mL^−1^	[257]
	*Escherichia coli*	50 CFU mL^−1^	10^2^−10^9^ CFU of UPEC mL^−1^	[258]
	Dopamine	9.5 nM	0.033–1 µM	[172]
Impedance spectroscopy	Human epidermal growth factor receptor 2	7400 pg mL^−1^	10–110 ng mL^−1^	[159]
	CA19-9	0.35 U mL^−1^	Not reported	[137]
	Prostate-specific antigen	0.48 pg mL^−1^	1–10,000 pg mL^−1^	[259]
	Sequence specific to chronic myelogenous leukaemia	1 fM	10^−15^–10^−6^ M	[177]
	2,4-dichlorophenoxy acetic acid	0.3 ppb	1–100 ppb	[260]
Transistor	SARS-CoV-2 spike protein	0.55 fg mL^−1^	0.0055–5.5 pg mL^−1^	[180]
	SARS-CoV-2 nucleocapsid protein	0.016 fg mL^−1^	0.016–16 pg mL^−1^	[180]
	Microcystin-LR	0.6 pg mL^−1^	0.001–1 ng mL^−1^	[244]
	miRNA-15	0.03 fM	0.1 fM–10 nM	[261]
	SARS-CoV-2 S1 antigen	4.12 fg mL^−1^	0.1 fg mL^−1^–5.0 pg mL^−1^	[28]
	Glucose	0.01 mM	0.01–2 mM	[262]
	influenza A virus DNA	1 pM	1 pM to 10 nM	[263]
	HIV-1 Tat protein	600 pM	0.2 nM–1μM	[264]
	cDNA	0.88 μg L^−1^	1.6 × 10^−4^–5 μmol L^−1^	[265]

## Data Availability

Not applicable.
